# Optimising psychological treatment for Anxiety DisordErs in Pregnancy (ADEPT): study protocol for a feasibility trial of time-intensive CBT versus weekly CBT

**DOI:** 10.1186/s40814-021-00838-8

**Published:** 2021-04-30

**Authors:** Fiona L. Challacombe, Laura Potts, Ben Carter, Vanessa Lawrence, Alaina Husbands, Louise M. Howard

**Affiliations:** Institute of Psychiatry, Psychology & Neuroscience, King’s College London, De Crespigny Park, London, SE5 8AF UK

**Keywords:** Perinatal, Anxiety, Pregnancy, Treatment, Cognitive behaviour therapy, Feasibility, OCD, Panic disorder, Post-traumatic stress disorder, Social phobia

## Abstract

**Background:**

Moderate to severe anxiety disorders such as obsessive-compulsive disorder (OCD), post-traumatic stress disorder (PTSD), social phobia and panic disorder are common, and affect approximately 11–16% of women in pregnancy. Psychological treatments for anxiety disorders, primarily cognitive behaviour therapy (CBT), have a substantial evidence base and recently time-intensive versions have been found as effective as weekly treatments. However, this has not been trialled in women who are pregnant, where a shorter intervention may be desirable.

**Methods:**

The ADEPT study is a feasibility randomised controlled trial with two parallel intervention groups. Time-intensive one-to-one CBT and standard weekly one-to-one CBT delivered during pregnancy will be compared. Feasibility outcomes including participation and follow-up rates will be assessed, alongside the acceptability of the interventions using qualitative methods.

**Discussion:**

The study will provide preliminary data to inform the design of a full-scale randomised controlled trial of a time-intensive intervention for anxiety during pregnancy. This will include information on the acceptability of time-intensive interventions for pregnant women with anxiety disorders.

**Trial registration:**

10.1186/ISRCTN81203286 prospectively registered 27/6/2019.

## Background

The mental health of pregnant women and those with a baby up to 1 year (known as the perinatal period) is a priority due to the potential impact on both mother and child [1]. Anxiety disorders are common and functionally impairing, affecting 11–16% of women in pregnancy and 8–17% of women in the postpartum period [2–5]. Pregnancy can exacerbate or trigger anxiety disorders and can also elicit new fears related to pregnancy and parenting [6]. For most women, antenatal anxiety disorders persist into the postpartum period [4, 7], and they can increase the risk of postpartum depression [8]. Postpartum anxiety disorders have been associated with impaired maternal functioning [9–12], excessive infant crying and feeding problems including lower rates of breastfeeding. Children of perinatally anxious mothers are at raised risk of experiencing emotional and behavioural problems [13–16].

It is therefore important to identify and treat antenatal anxiety disorders quickly, to ameliorate or prevent these short- and long-term outcomes for both mother and child. Shorter exposure of the developing fetus to the elevated levels of maternal cortisol associated with anxiety may protect the child whilst successful antenatal treatment would also improve quality of life for the mother and potentially reduce the impact on parenting. The need for timely treatment is reflected in clinical practice guidelines [17]. However, the National Institute for Health and Care Excellence (NICE) guidelines for perinatal mental health [17] highlight the need for more research on treating moderate to severe anxiety disorders, in particular obsessive-compulsive disorder (OCD), panic, post-traumatic stress disorder (PTSD) and social phobia (recommendation 7.7.2.4). Each of these disorders can be triggered or exacerbated in the context of pregnancy and can affect mother-infant interactions and parenting [18].

Pregnant women prefer psychological treatments to pharmacotherapy, but there are barriers to such interventions including having time to attend sessions [19]. UK primary mental health care sites have reported dropout rates prior to treatment for perinatal women of up to 40% [20]. Shorter, but more intensive treatment could therefore be an innovative and welcome format that may improve engagement and optimise outcomes for both mother and baby.

Psychological treatments for anxiety disorders, primarily cognitive behaviour therapy (CBT), have a substantial evidence base [21–23]. Women are usually offered one-to-one disorder specific CBT based on best available evidence [24]. Individual CBT is ‘semi-idiographic’, that is, based on shared principles but tailored to the individual context and needs of the client. Modifications may be made according to the client’s physical state, such as not running upstairs in a panic exposure exercise if the person is unable, instead identifying other means to test relevant beliefs. Such modifications in pregnancy have been suggested and there is some evidence for the use of exposure-based CBT during pregnancy.

CBT is usually delivered in approximately 12-h-long weekly sessions, depending on the presenting disorder. Time-intensive CBT treatments (IN-CBT) have been trialled in (non-perinatal) patients with OCD, PTSD, social phobia and panic disorder with equivalent outcomes to standard weekly treatments, achieved in a much shorter time frame of 1 to 2 weeks [25–28]. This format has been found acceptable to patients. IN-CBT for postpartum OCD has been found to be safe and effective in the reduction of maternal OCD symptoms and acceptable to mothers [29, 30]. Given the clear aim of delivering fast and effective treatment, ideally before the baby is born, IN-CBT could be a helpful modification to traditional weekly CBT for pregnant women with anxiety disorders and may be more beneficial in terms of longer-term outcomes. Specific evidence is therefore needed to establish whether IN-CBT approaches are acceptable, produce equivalent engagement and adherence with treatment during pregnancy, are efficacious during pregnancy and helpful for maternal symptoms and parenting in the postpartum.

## Study aims and objectives

The primary objective of this research is to assess the feasibility of a definitive trial of antenatal IN-CBT compared with standard weekly antenatal CBT (treatment as usual) for women experiencing OCD, PTSD, social phobia or panic disorder in pregnancy. These disorders have evidence for the use of intensive approaches. Outcomes will assess the feasibility and acceptability of recruitment methods; recruitment rates and participants’ willingness to be randomised; the acceptability of assessment measures, intervention mode and intervention delivery; treatment fidelity; follow-up rates; and estimates of sample size parameters. We will use this information to inform a full-scale randomised controlled trial (RCT).

The secondary objectives are to collect and summarise clinical outcomes and physical and mental health service use for health economic evaluation.

## Methods

### Study design

This study is a feasibility 1:1 randomised controlled trial with two parallel intervention groups. Methods are presented as per the Standard Protocol Items: Recommendations for International Trials (SPIRIT) [31]. All elements of the SPIRIT checklist are reported below.

### Participants, interventions and outcomes

#### Study setting

The study is set within adult mental healthcare services in English National Health Service (NHS) settings. As the national publicly funded healthcare system for England, the NHS provides healthcare for all legal residents in the UK, and mental healthcare is amongst the services free at the point of use.

The study setting will be four outpatient Improving Access to Psychological Therapies (IAPT) services within South London and Maudsley (SLaM) NHS Foundation Trust in South East London. The four outpatient IAPT services (Lambeth, Lewisham, Southwark and Croydon) have a named perinatal lead or experienced therapist who will provide standard weekly and IN-CBT treatments within the trial. The Centre for Anxiety Disorders and Trauma (CADAT) is part of Lambeth, Lewisham and Southwark IAPT services and will provide both treatments.

### Participant recruitment and eligibility criteria

Women will be recruited to the trial in one of 2 ways:

- Women attending antenatal booking clinics in a South East London maternity service (in the boroughs of Lambeth, Lewisham, Southwark or Croydon only) may be approached by their midwife with information about the study, who may contact the researchers on their behalf if the woman is interested and agrees. Alternatively, women may self-refer to the trial if they feel they are experiencing symptoms of anxiety consistent with one of the anxiety disorders being treated. Advertisements (e.g. leaflets and posters) for self-referral to the trial will be positioned in maternity service waiting rooms and GP surgeries and other settings for local pregnant women, e.g. children’s centres to encourage self-referral.
- Women may be referred to the trial by a therapist in the IAPT services if the therapist assessed the woman as having an anxiety disorder and potentially suitable for the trial. Clinicians will be regularly reminded about the study and flyers and posters made available.

### Inclusion criteria

Women must have the following characteristics prior to randomisation:

1. Aged ≥ 18 years
2. Pregnant, between 12 and 25 weeks gestation
3. Meet criteria for Diagnostic and Statistical Manual of Mental Disorders Fifth Edition (DSM-V) for OCD, PTSD, social anxiety or panic disorder on the Structured Clinical Interview DSM-V (SCID-V)
4. Eligible for referral to Lambeth, Lewisham, Southwark or Croydon IAPT services (i.e. has local general practitioner, common mental disorder and level of risk manageable within primary care)
5. Available (by self-report) for either IN-CBT or standard weekly treatment
6. Not taking psychotropic medication or taking stable dose of medication for at least 6 weeks
7. Able to provide informed consent

### Exclusion criteria

The exclusion criteria are as follows:

1. Pregnant women with a primary DSM-V depressive disorder, affective or psychotic disorder or current problems with substance abuse
2. Pregnant women with ‘complex PTSD’ (defined as prolonged multiple traumas affecting a number of domains)
3. Pregnant women who have a medically high-risk pregnancy at the time of recruitment involving significant additional management (e.g. multiple sclerosis, lupus, polycystic ovary syndrome)
4. Pregnant women who are receiving cognitive behaviour therapy or another individual or group psychological therapy
5. Pregnant women who are unable to read English adequately to complete questionnaires

**Fig. 1:**
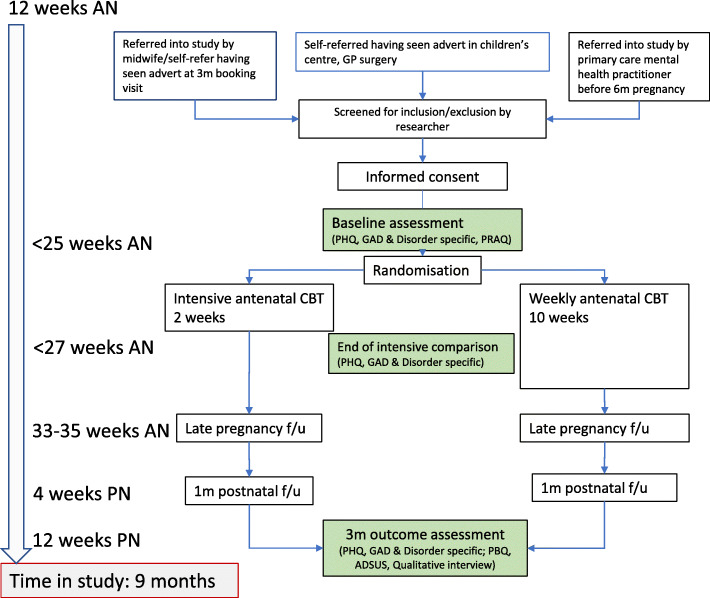
Schematic representation of time in the study

The research psychologist will obtain informed consent from potential trial participants who will send a signed consent form. Women will be given at least 24 h after receiving the information sheet to decide whether to proceed with consent and baseline measures. Figure 1 shows the participant timeline through the study.

## Assignment of interventions

### Sequence generation

Participants will be randomly allocated using a 1:1 ratio to (1) time-intensive CBT (IN-CBT) or (2) standard weekly CBT (i.e. treatment as usual). Participants in either allocation group will receive treatment delivered by a high-intensity cognitive behaviour therapist (HI CBT therapist). Randomisation will be conducted via an independent online system based at the King’s Clinical Trials Unit (King’s CTU) based at King’s College London. Random allocation will be at the level of the individual participant and minimisation will be used to achieve balance amongst minimisation factors; disorder (OCD, panic, PTSD, social phobia) and site (Croydon, Lambeth, Lewisham, Southwark).

### Concealment mechanism

Participants will be randomised to the trial groups (1:1 allocation) to ensure allocation concealment is high. Any preference on allocation will be recorded prior to randomisation.

### Implementation

The allocation sequence is generated by the KCTU online system. The research psychologist will enrol participants. Once an eligible woman has given consent to participate in the study and completed baseline measures, the research psychologist will use the online system to perform the randomisation. The research psychologist will communicate information about the allocation group to the participant (either time-intensive CBT treatment (IN-CBT) or standard weekly CBT treatment) and to the therapist who will arrange an appointment with the participant at the earliest mutually convenient opportunity.

### Blinding

For each allocation to the IN-CBT or standard weekly treatment group, the participant, their therapist and the research psychologist will be aware of the allocation. The research assistant will be blind to the group allocation and will not have access to allocation databases. The research assistant will collect outcome measures at baseline, 1 month and 3 months (primary outcome point) and will be blind. They will conduct the qualitative interview last as it will likely lead to unblinding. The senior statistician will be blind to the group allocation until the end of the trial.

The senior statistician will become unblind once the primary and secondary analyses have been performed. The Statistical Analysis Plan will be drafted by the trial statistician and the senior statistician will contribute fully blinded. The outcome assessor will be unblinded at the end of final outcome assessments by conducting the qualitative interview, as the study is not double blind. This occurs after the other assessment information has been collected.

### Data collection and management

#### Baseline assessment

Baseline measures will be collected by the research psychologist or research assistant prior to the assignment of participants to the intervention groups. Baseline measures will be collected via weblink using existing data collection methods in IAPT services or hard copies as preferred by participants and will be completed in participants own time. Initial clinical interviews will be conducted over the phone at a suitable time for the mother, or in person if she prefers. As per standard practice in IAPT services, anxiety, depression and disorder specific measures will be collected at each point of contact. Participants will be asked to complete the additional measures at the baseline and outcome points. The postnatal follow-up video and qualitative interview (see below for details) will be collected during a visit to the participant’s home by the research assistant who will be blind to the allocation status of the participant. Assessments may take place via phone or video link using Microsoft Teams, depending on current restrictions due to the Coronavirus.

A list of measures collected during the study and the timepoints of data collection are presented in Table 1.

**Table 1:** Study measures at each timepoint

Measure	Pre-treatment baseline assessment	After first 2 h of treatment	Late pregnancy follow-up session^a^	3m postnatal outcome assessment
Screening measures				
Structured clinical interview for DSM-V (SCID)	X			
Routinely collected IAPT measures (at every clinical session)				
Demographics	X			
PHQ-9 (Depression)	X		X	X
GAD-7 (Anxiety)	X		X	X
The Work and Social Adjustment Scale (WSAS)	X		X	X
Disorder specific measure (OCI, Panic, IES, SPIN)	X		X	X
Trial specific measures				
Working Alliance Inventory (WAI)		X		
Pregnancy related anxiety questionnaire (PRAQ)	X		X	
Postpartum bonding questionnaire (PBQ)				X
Mother-infant interactions (CARE index)				X
Adult Service Use Measure (ADSUS)				X
Qualitative interview				X

^a^One hour follow-up session with therapist administered during late pregnancy (33–35 weeks gestation)

#### Measures related to primary objectives

The primary feasibility outcomes will be determined through analysis of the recruitment and follow up data. Therapist time, including participant contact and assessment of time spent in other indirect participant related activities (e.g. supervision, training, administration etc.), will be recorded by therapists using standardised clinical notes. Therapy content and adherence will be determined by analysis of these notes.

As is standard practice in CBT, therapy sessions will be audio-recorded by therapists. A random selection of therapy tapes from each therapist will be rated by assessors blind to the aims of the study using a standardised measure of cognitive therapy skills, the Cognitive Therapy Rating Scale (CTS-R [32];).

### Measures related to secondary objectives

#### Initial clinician interview collected at baseline

*Structured clinical interview for DSM-V* (SCID-V [33];). This semi-structured interview is used by clinicians to establish DSM-V diagnoses, which will be part of initial screening for inclusion in the trial [34].

Routinely collected IAPT data collected at baseline, each clinical session and 3 months postpartum:

1. *Demographic* (at baseline only)—maternal age, ethnicity, occupation, relationship status, whether they are taking psychotropic medication and pregnancy stage will be recorded.
*GAD-7 [35] is a well validated and widely used 7 item measure of anxiety and a key IAPT outcome measure used routinely. A change of 4 or more on the GAD has been found to be clinically significant across anxiety disorders [36]. *This is the potential primary outcome measure for a future evaluation trial.
3. *PHQ-9* [37] is a well-validated 9-item questionnaire used to measure symptoms of depression. It is also a key IAPT outcome measure.
4. The *Work and Social Adjustment Scale* [38] is a 5-item patient self-report measure, which assesses the impact of a person’s mental health difficulties on their ability to function in terms of work, home management, social leisure, private leisure and personal or family relationships.
5. Disorder specific measures: one of the following will be used depending on primary disorder.

- OCD: Obsessive Compulsive Inventory-Revised (OCI [39];). This is a 42-item self-report inventory concerning symptoms of OCD. The internal consistency for the full scale is high (0.86–0.95). The OCI also shows good discriminative validity and is reliable to measure change in symptoms over time.
- Panic: Mobility Inventory (alone) [40]. This self-report scale requires respondents to rate avoidance of a range of specific situations over the last week on a scale of 0–4. The inventory has good reliability, validity and sensitivity to change.
- PTSD: Impact of Events Scale (IES [41]). This is a 22 item self-report scale of symptoms of PTSD. The scale has excellent internal consistency [42].
- Social Phobia: The Social Phobia Inventory (SPIN [43]) has 17 items and a cut-off score of 19 or above. For each item, respondents rate from 0 to 4 how bothered they have been by the item during the past week. The total score provides a measure of the severity of social phobia and the item has good sensitivity to change.

### Trial-specific measures

Working alliance inventory–Short Revised–collected after 2 h of treatment

6. Working Alliance Inventory–Short Revised [44]. This 12-item questionnaire will be completed by therapists and participants in both arms.

Pregnancy Anxiety–collected at start of treatment and late pregnancy follow-up session

7. Pregnancy-Related Anxiety Questionnaire (PRAQ [45];)*.* This 10-item self-report questionnaire assesses anxiety related to childbirth and will be administered at baseline and the last antenatal appointment treatment session. The pregnancy-related anxiety scale has acceptable internal reliability (Cronbach’s α = .78).

Infancy related measures—collected at 3 months postpartum

8. Postpartum Bonding Questionnaire (PBQ) [46]. This self-report instrument consists of 25 items to be rated on a scale of 0 (‘never’) to 5 (‘always’) and assesses the maternal perception of her felt bonding with the infant.
9. Mother infant interactions

Mother-infant interactions at 3 months postpartum will be captured in a 3-min video taken during play and nappy change at home and subsequently assessed by a trained rater using the CARE Index [47]. This tool has been widely used in scientific research with mothers that have mental health difficulties as early as 1 to 5 months postpartum [48–50] and validated for use with families from different social classes and cultural backgrounds [51]. Coding comprises seven aspects of adult and infant dyadic behaviour: four aspects concentrate on affect (facial expression, verbal expression, affection and body contact) and three focus on temporal contingencies (turn-taking, control and developmental appropriateness of chosen activity). Each aspect of adult and infant behaviour is evaluated individually and summed to make seven scale scores. For adults, these are sensitivity, unresponsiveness and controlling. Infants (birth to 15 months of age) are coded on cooperativeness, difficultness, compulsivity and passivity. Interactions receive a score on each aspect of adult and infant behaviour and scores are then summed to create the seven scale scores, each on a range from 0 to 14 [47]. For example, a ‘sensitive dyad’, the mother must achieve a score of 11 or higher on the sensitivity scale. A score of 7 or more is required to rate the interaction as ‘adequate’. Five to 6 points mark the ‘inept’ range and suggest the need for parental education. Four or fewer points are considered as in the ‘high risk’ range, implying risk of abuse or neglect. Coding of the interaction takes between approximately 30–40 min. Video-taped interactions will be coded by trained, reliable coders, as recommended by Crittenden [47]. To reduce measurement bias, the coders will be unaware of the hypotheses that are being tested in the study and blind to the mental health status of the women.

Health economic measures collected at 3 months.

10. Adult Service Use Measure (ADSUS, unpublished [52]). The AD-SUS is a measure of resource use developed for use in mental health populations and a version adapted for use in prenatal populations as part of an NIHR PGfAR will be used (the ESMI study). This measure will be used to capture maternal and child utilization of all health and social care services (including health visitor, GP visits).

Measures 8–10 have been included to assess the potential impact on parenting and service use.

### Qualitative data

Qualitative interviews will be conducted with an estimated 15–20 women undergoing IN-CBT and 15–20 women undergoing standard CBT, or until inductive thematic saturation is reached, and new data is easily accommodated within the thematic framework to further examine the acceptability and feasibility of the intervention and study design [53]. Interviews will explore experiences of having an anxiety disorder in pregnancy, experiences of treatment including perceived benefits and limitations and challenges during treatment and in postpartum adjustment. Experiences of recruitment, randomisation and assessments will also be examined. Topics will be derived in collaboration with the project advisory group. Purposive sampling will be conducted on the basis of diagnosis, socio-economic status, ethnicity, recruitment source and session attendance to explore a range of perspectives. Data collection and analysis will be conducted in parallel with the topic guide and sampling strategy amended iteratively to explore the diversity of data. Interviews will take place at the final outcome point 3 months postpartum. These will be recorded and transcribed. All therapists will also be interviewed to examine how IN-CBT and standard CBT were delivered in practice (e.g. treatment fidelity, ease of delivery, perceived engagement).

### Details of interventions

Cognitive behaviour therapy interventions are the NICE guideline recommended treatment for anxiety disorders and are considered effective [21–23]. Psychological interventions are generally preferred by pregnant women and this study seeks to investigate a time-intensive approach given the time parameter of pregnancy. Therefore time-intensive and standard weekly CBT approaches will be compared.

There are well developed specific protocols and techniques for using CBT to treat anxiety disorders [54]. In general, CBT follows the course of establishing the participant’s goals for therapy and developing a patient-specific formulation based on the relevant model (e.g. Salkovskis for OCD; Wells & Clark for Social Phobia; Ehlers & Clark for PTSD; Clark for Panic disorder). The formulation identifies relevant beliefs and behaviours that maintain the anxiety disorder. Cognitive and behavioural strategies are then used to help patients change the beliefs and behaviours that maintain their anxiety disorder. A standard modification to treatment in the perinatal context would be to include planning and support for the demands of the postpartum environment whilst the person is attending antenatal treatment. All participants and therapists audio-record sessions (as per standard clinical practice) to listen to between appointments and participants will be encouraged to apply new learning and strategies as homework. The content of treatment will be the same for both intervention arms.

#### Time-intensive CBT

This will comprise 8–10 h (depending on the disorder) of one-to-one CBT delivered in 4–5 sessions over 1–2 weeks, delivered at the earliest convenient point between 12 and 36 weeks of pregnancy. The CBT treatment will be delivered by a high-intensity cognitive behaviour (HI CBT) therapist, which is standard practice within IAPT services. Two follow up sessions of 1 h each will then be offered by the HI CBT therapist which will include one in late pregnancy (33–35 weeks’ gestation) and one at 1m postpartum.

#### Standard weekly CBT (treatment as usual)

Standard weekly CBT will comprise 8–10 hours (depending on the disorder) of one to one CBT on a one hour per week basis. This is termed ‘high intensity’ CBT in IAPT services and will be offered to all women who participate in the trial and are randomised to standard weekly CBT. The CBT treatment will be delivered by a High Intensity Cognitive Behaviour (HI CBT) Therapist, which is standard practice within IAPT services. Two follow up sessions of one hour will then be offered by the HI CBT Therapist which will include one in late pregnancy (33–35 weeks’ gestation), one at 1m postpartum.

Treatments may take place via phone or video link using Microsoft Teams, depending on current restrictions due to the Coronavirus.

### Provisions for post-trial care

Women requiring and wishing for further intervention after the end of the trial will be referred or signposted to an appropriate intervention. This may be for further psychological therapy within IAPT or within more specialised perinatal mental health services.

### Criteria for discontinuing or modifying allocated interventions

Adverse events will be monitored and recorded including the mental and physical health of the mother and any pregnancy-related events. Should participants deteriorate clinically in terms of significantly increased symptoms of anxiety or depression, or an increase in risk to themselves or others, then the intervention will be stopped and a referral made to specialist perinatal services and/or other services as clinically appropriate.

### Strategies to improve adherence to interventions

Fidelity will be defined as delivering all key treatment components during treatment and will be established by a checklist of key components of treatment derived from core competencies for specific disorders using treatment notes [23]. This will be completed by the therapist at the end of each session. Standardised therapy note sheets will be kept and checked to establish fidelity against the checklist. Therapists will also be required to submit a random selection of therapy recordings to be rated for fidelity to CBT on an established scale, the Cognitive Therapy Rating Scale (CTS-R).

Client engagement and adherence to therapy will be rated by therapist at each session on a 4-point Likert scale, number of sessions attended will be recorded and the participants’ perspective will be assessed in detail in a qualitative interview at 3 months.

### Outcome progression criteria

Primary feasibility outcomes:

(i) if it is possible to identify/recruit patients via (a) information provided in healthcare settings at booking visits/by midwives/self-referral and (b) approach by a primary care therapist. We require a minimum of 30% of those approached to be eligible participants from each recruitment method for each to be deemed feasible. An acceptable recruitment rate (number of consented participants) would be at least 3 participants/month
(ii) If participants are willing to be randomised. We require that 70% of eligible participants are randomised to be deemed feasible
(iii) If the intervention is received as intended in both arms using a fidelity content checklist. A minimum of 70% of participants in the trial would need to complete > 60% of each intervention in hours for each to be deemed feasible, i.e. 7.2 h out of 12 [28]. For completers in the intensive arm, these treatment hours will need to be completed in the 2-week window
(iv) Acceptability of both interventions to participants will be determined by qualitative investigation enquiring about experiences and positive and any negative effects of all aspects of treatment, and a 3-point rating scale to assess how useful the treatment was for anxiety and parenting
(v) Establishing the parameters needed in order to estimate the sample size for a full trial. For example, standard deviation of the potential primary outcome measure GAD-7
(vi) Participation and data completion at 3m outcome assessment; a follow up rate of > 70% is required to determine feasibility
(vii) The acceptability of assessment measures to participants; this will be determined by qualitative interview and brief 3-point rating scales asking if it was acceptable and clear

Secondary clinical outcome measures:

(i) Clinical outcome measures for depression, anxiety, overall functioning and anxiety disorder symptoms collected at 3 months postpartum
(ii) Health service use at 3 months postpartum

### Plans to promote participant retention and complete follow-up

Participants will receive an incentive (£10) for the additional burden incurred from completing assessments at two time points. Participants will receive £10 for participating in the baseline assessment and £10 for participating in final outcome point assessments. They will be encouraged to take part in the final outcome point assessment regardless of treatment completion.

### Sample size estimation

In line with guidance on feasibility studies, no power calculation has been carried out [55]. A sample size of 30 in each intervention group has been recommended to answer questions relating to feasibility [56, 57].

### Data management

The trial statistician will assist with management of the trial data which will be split into 3 databases. The participant main database and therapy database will be stored in separate SPSS files on a network drive only accessible by the CI and trial statistician. The AE (adverse events) database will be stored as a password-protected Excel file on a network drive accessible by the CI only. All databases will be backed up periodically (approx. every 3 months), by creating a date-stamped ZIP file storing all databases and storing in a subfolder titled ‘Archives’. This will be carried out by both the trial statistician and the CI separately.

All research data will be pseudonymised using unique identification numbers and stored without contact details (names or addresses). Associations between participants' contact details and identification numbers will be stored in a separate encrypted electronic password-protected database. Access to this document will be restricted to the Chief Investigator. All data will be held on a secure database on an encrypted, password-protected computer and access to it will be restricted to the research team. Audio files of the qualitative interviews will be retained until they have been transcribed to written form. Transcriptions of qualitative interviews will be completed as soon as possible after collection, anonymised and uploaded to the computer software programme, QSR N-VIVO. Hardcopies of study consent forms held by the central research team will be kept in a locked cabinet at the Institute of Psychiatry, Psychology & Neuroscience and retained for 7 years post research data analysis.

#### Questionnaire data

Questionnaire data will be collected via weblink using existing data collection methods in IAPT services and/or hard copies as preferred, completed in participants own time. Initial clinician interviews will be conducted over the phone at a suitable time for the mother, or in person if she prefers. IAPT measures are routinely collected at each contact so collecting this treatment data will not involve additional burden for women.

All outcomes will be summarized at 3 months postpartum; video and qualitative interview audio data will be collected during a visit to the participant’s home (or remotely) at this point in addition to the IAPT measures.

As per standard practice in IAPT services, anxiety, depression and disorder-specific measures will be taken at each point of contact. Participants will be asked to complete the additional measures at the baseline and outcome points.

#### Video data

All video recordings will be collected using password-protected iPads or via Microsoft Teams. The iPads will be brought back to the university site for secure storage directly after the interview. Video recordings will be immediately uploaded on to the secure university computer network, which is password-protected and which only the research team have access. Once the video recordings have been transferred onto the secure computer network, they will be permanently deleted from the iPads or TEAMS. In order to verify the deletion of the files, we will empty the ‘trash’ file of the iPads. The iPads will be linked to a cloud account in order to be able to wipe them remotely should they be stolen. The video data files will be saved on the secure university computer network using only participant ID and date as identifiers, and only researchers involved in the project will have access to the drive with the video recordings.

### Confidentiality

Participants will be registered as patients under secure local NHS clinical patient records systems (known as IAPTUS) which will hold their personal data. Participants research data (questionnaires) will be pseudonymised and entered on password-protected databases as described above, kept on university computers. Video and audio data will be identified by participants number only. Participants names will be kept in a separate password protected database.

### Statistical methods

#### Quantitative data

Quantitative data analysis will be primarily descriptive to aid the planning of a future RCT. Participant flow through the study will be presented following CONSORT guidelines. Descriptive data will be presented in the form of means and standard deviations; medians and ranges; or percentages with 95% confidence intervals, as appropriate depending on the data being described.

The following feasibility parameters will be calculated: (1) percentage of participants meeting eligibility criteria (those eligible/those approached or referred) by recruitment channel into the study; (2) number of participants recruited per month (defined as those consented); (3) percentage of individuals consenting to randomisation (those randomised/those consented); (4) percentage of participants completing treatment in each arm (number of participants receiving adequate treatment dose, defined as 60% of treatment hours/those randomised); (5) percentage completing the outcome measures at 3m postpartum (number of participants completed at least one follow-up measure/those randomised); (6) between group pre-post effect sizes and confidence intervals, adjusting for randomisation stratifiers (fixed effect) and therapist (random effect), and variance on the potential primary outcome measure at 3 months post-randomisation; (7) summary statistics on acceptability of intervention and outcome measures measured using a 3-point Likert scale. Data will be presented overall and subgroup summaries by disorder.

Descriptive data on therapist competence and compliance will be presented using standardised Cognitive Therapy Scale Ratings (CTS-R) and number of treatment elements completed from checklist.

### Qualitative data

Focussed thematic analysis will be utilised, as in a previous study evaluating experiences of treatment [58, 59] which compared participants’ experiences in a non-randomised trial of intensive and weekly CBT for OCD. Constant comparison method [60] will be used to delineate themes and sub-themes relating to participants’ experiences and attitudes towards treatment.

Two researchers will independently code three transcripts to help identify and discuss alternative interpretations of the data [61]. An analytical framework will be constructed around the perceived value, acceptability and feasibility of the treatment, which will be applied to the remaining transcripts, with themes and subthemes refined as necessary. Deductive codes will be supplemented with inductive codes to reflect the emergent priorities and concerns in the data. Ideas about themes and their relationships will be recorded in theoretical memos and discussed amongst our Project Service User Advisory Group. The computer programme QSR N-VIVO will be used to process the transcripts, enabling coding and retrieval of a large volume of narrative data.

## Monitoring

### Composition of the coordinating centre and trial steering committee

The Trial Management Group (TMG) comprises FC, LP, BC and VL. An independent trial steering and data monitoring committee will be established to examine the clinical progress of trial participants and the conduct of the trial. The group comprises two clinicians, one statistician and one expert by experience. This will meet soon after the beginning of recruitment and then every 6 months to oversee the trial, check data and adverse events; a trial report is prepared in advance. A charter for the group is available from the corresponding author.

### Patient and public involvement

Patients were involved in advising on the design of the study. A patient advisory group (PAG) comprising 4–6 women with lived experience of perinatal mental health problems will be set up to advise on the study and will meet approximately annually.

### Adverse event reporting and harms

Participants will be carefully monitored throughout treatment by asking them for relevant information at each contact. Data will be collected from participants on potential adverse effects including pregnancy outcomes and prematurity.

Adverse events will be monitored and recorded, including the mental and physical health of the mother and any pregnancy-related events. Should participants deteriorate clinically in terms of significantly increased symptoms of anxiety or depression, or an increase in risk to themselves or others, then the intervention will be stopped and a referral made to specialist perinatal services and/or other services as clinically appropriate.

### Dissemination plans

1. Results of the trial will be fed back to participants via a newsletter.
2. Findings from the study will be published in a series of high-quality peer reviewed papers. These will include journals targeted at academics, CBT practitioners, perinatal specialists and health service managers.
3. Study results will be presented at academic and service user led conferences as well as conferences for managers. Examples would be the British Association for Behavioural and Cognitive Psychotherapies conference, Marce conference, Maternal Mental Health Alliance conference.
4. Findings from the study will be disseminated to service user groups in perinatal mental health and for anxiety disorders and umbrella organisations such as the maternal mental health alliance.
5. Clinical approaches developed from the study would be disseminated in clinical skills workshops and training for existing and new CBT therapists. These would take place in IAPT and specialist perinatal settings.

### Protocol version and amendments

This publication is based on protocol number and date ADEPT Protocol v3_03.06.20.

## Discussion

There is a need for effective and accessible treatments for pregnant women with anxiety disorders. This study will be the first to examine the use of time-intensive CBT in pregnant women. It is designed to assess questions of acceptability and feasibility, in order to inform the design of a full-scale RCT. The study data will inform this using both quantitative and qualitative data to assess feasibility outcomes. Time intensive CBT has the potential to be an effective and time efficient intervention for women with anxiety during pregnancy. It is designed to be delivered in existing psychological services and could therefore potentially be rolled out widely across the NHS.

### Trial status

Recruitment started in September 2019 and is expected to be completed by end September 2021.

## Data Availability

Not currently applicable. Participant level data will not be available due to the small and potentially identifiable dataset.

## Abbreviations

ADSUS: Adult service use measure
CADAT: Centre for anxiety disorders and trauma
CBT: Cognitive behaviour therapy
CI: Chief investigator
CTS-R: Cognitive therapy scale-revised
CTU: Clinical trials unit
DSM-V: Diagnostic and statistical manual of mental disorders fifth edition
GAD: Generalised anxiety disorder
GAD-7: Generalised anxiety disorder scale-7 items
HI: High intensity
IES: Impact of events scale
NHS: National health service
NICE: National institute for health and care excellence
IAPT: Improving access to psychological therapies
OCD: Obsessive-compulsive disorder
OCI-R: Obsessive compulsive inventory-revised
PAG: Patient advisory group
PBQ: Postpartum bonding questionnaire
PHQ-9: Patient health questionnaire
PRAQ: Pregnancy-related anxiety questionnaire
PTSD: Post-traumatic stress disorder
RCT: Randomised controlled trial
SCID-V: Structured clinical interview DSM-V
SLaM: South London and Maudsley NHS Trust
SPIN: The social phobia inventory
SPSS: Statistical package for the social sciences
WSAS: Work and social adjustment scale
TMG: Trial management group
